# A Scoping Review of Vicarious Trauma Interventions for Service Providers Working With People Who Have Experienced Traumatic Events

**DOI:** 10.1177/1524838021991310

**Published:** 2021-03-09

**Authors:** Jeongsuk Kim, Brittney Chesworth, Hannabeth Franchino-Olsen, Rebecca J. Macy

**Affiliations:** 1 School of Social Work, 2331 University of North Carolina at Chapel Hill, NC, USA; 2 Gillings School of Global Public Health, 2331 University of North Carolina at Chapel Hill, NC, USA

**Keywords:** vicarious trauma, intervention/treatment, domestic violence, memory and trauma, mental health and violence, treatment, PTSD

## Abstract

Health and human service providers who aid traumatized individuals frequently experience vicarious trauma (VT). Although VT plays a critical role in service providers’ mental health and well-being, as well as in the quality of their service provision, little information is available concerning the development and implementation of VT interventions for service providers. To advance the development of evidence in this area, we undertook a scoping review in which we reviewed existing interventions intended to address VT among service providers working with traumatized clients. Searches of electronic databases were conducted to identify studies published in peer-reviewed journals, with no date restrictions. Over 1,315 citations were reviewed, and a total of 27 studies were included in the final review. The findings show that VT interventions in the literature can be divided broadly into four categories: psychoeducation, mindfulness intervention, art and recreational programs, and alternative medicine therapy. The VT interventions reviewed generally showed promise in their key outcomes, including reductions in secondary trauma stress, compassion fatigue, burnout, and other mental health outcomes. However, the current body of research is lacking both in rigor and in specificity regarding the definition of VT. Furthermore, existing VT interventions are generally self-care based and tend to focus on general stress management rather than addressing the specific effects of VT. Therefore, we call for an increase in efforts to tailor VT interventions to different service settings and participant characteristics, as well as greater attention to developing primary VT interventions at the organizational level.

Health and human service providers working with individuals who have experienced trauma (e.g., abuse and assault, grief and loss, human trafficking, military combat, natural disasters, and terrorism) are at risk of developing vicarious trauma (VT) through repeated exposure to and empathic engagement with their clients’ and patients’ traumatic experiences (Raunick et al., 2015; Sprang et al., 2019). Even though service providers’ VT is a typical response to working with traumatized people (Figley, 1995; Molnar et al., 2017), such symptoms may negatively affect service providers themselves, resulting in a broad range of cognitive, emotional, and behavioral changes (Bercier & Maynard, 2015). Worryingly, VT symptoms may also affect service quality, causing a deterioration in provider decision making and service delivery (Branson, 2019; Schauben & Frazier, 1995).

In the last few decades, researchers and practitioners have developed different interventions to prevent and decrease service providers’ trauma-related symptoms (Bercier & Maynard, 2015; Molnar et al., 2017). However, little comprehensive information has been available regarding VT intervention research, including effectiveness and limitations (Bercier & Maynard, 2015; Molnar et al., 2017; Sprang et al., 2019). The scarcity of such research highlighted a critical need to examine and synthesize systematically existing VT intervention studies to help advance evidence development in this area (Molnar et al., 2017; Sprang et al., 2019).

## VT, Secondary Trauma Stress, and Compassion Fatigue

Broadly, VT refers to significant, indirect experiences of distress resulting from empathic engagement with clients who experienced trauma (World Health Organization, 2013). VT is conceptually linked to and overlaps with other two constructs, namely, secondary traumatic stress (STS) and compassion fatigue (CF) (Jenkins & Baird, 2002; Newell & MacNeil, 2010). VT, STS, and CF have been often used interchangeably (Newell & MacNeil, 2010; Rauvola et al., 2019) or grouped together as a broad construct (e.g., empathy-based stress) explaining service providers’ comprehensive trauma-related symptoms (Rauvola et al., 2019). Given that VT, STS, and CF share common causes and consequences for service provision, these terms are understandably used interchangeably. However, many researchers have urged the conceptual differentiation of these constructs and some researchers have identified them as symptomatically different constructs (Molnar et al., 2017; Newell & MacNeil, 2010; Rauvola et al., 2019).

Pearlman and Saakvitne (1995) defined VT as a change of cognitive schemas resulting from consistent empathic engagement with traumatized individuals (Newell & MacNeil, 2010). That is, indirect exposure to trauma changes service providers’ cognitive schemas and beliefs about the self, others, and the world (Cohen & Collens, 2013; Moulden & Firestone, 2007; Pearlman & Saakvitne, 1995). For example, a domestic violence shelter worker who regularly helps women who have been victimized by their male partners may start to believe that all men will become violent (Molnar et al., 2017). While VT referred to a process of cognitive change, the construct of STS placed more emphasis on the outward symptoms (Figley, 1995). The symptoms of STS were similar to the full range of post-traumatic stress disorder (PTSD) symptoms, including intrusive thoughts, insomnia, chronic irritability or angry outbursts, fatigue, difficulty concentrating, avoidance of clients, and client situations (Figley, 1995; Jenkins & Baird, 2002). That is, service providers exposed to indirect trauma might show the same PTSD symptoms experienced by individuals directly exposed to trauma. In the most recent version of the *Diagnostic Statistical Manual* (the DSM-5), the PTSD diagnosis now includes an expanded definition of trauma that includes those who have experienced secondary trauma in their professional role (American Psychiatric Association, 2013). Figley (1995) later changed the name of STS to CF, which applied to broader populations and goes beyond those professions where compassion is an expected service (Ludick & Figley, 2017). CF was best defined as a syndrome consisting of a combination of STS symptoms and professional burnout (Figley, 1995; Newell & MacNeil, 2010).

Put together, initially, VT-related symptoms seem to be defined as different concepts. However, the boundaries remain ambiguous and much of the literature still use these constructs interchangeably (Bercier & Maynard, 2015; Sprang et al., 2019). Thus, though we recognize their different natures, our review on interventions related to preventing or treating VT included not only VT intervention programs but also STS and CF interventions, which allowed us to achieve a comprehensive review of existing VT interventions. However, for readers’ ease, we called all of the interventions addressing service providers’ VT, STS, and CF “VT intervention” here.

## Previous Reviews on VT Interventions and the Current Study

While diverse interventions to prevent and ameliorate service providers’ VT-related symptoms have been developed and studied, there have been limited efforts to date to review this research (Rauvola et al., 2019). For example, Everly (1999) explored the effectiveness of group psychological debriefings on VT with emergency care providers through a meta-analysis. However, this review only focused on a particular type of VT interventions (i.e., debriefing) and, thus, did not review a range of relevant literature. In addition, the review included not only emergency workers but also trauma victims in four sample studies, without a specific rationale.

Bercier and colleagues (2015) attempted to conduct a systematic review of studies on interventions for STS with mental health workers. However, no studies met their inclusion criteria for full-text review, given issues with study design (nonrandomized experimental or quasi-experimental design) or participant characteristics (nonmental health workers). Consequently, they concluded that the existing evidence lacked the rigor necessary for a review and called for renewed evaluation efforts with stronger research methods (Bercier & Maynard, 2015). Recently, Sprang and colleagues (2019) attempted to review evidence from STS interventions and to convene meetings of STS experts in order to discuss best practices in treatment approaches and to consider strategies for moving the field forward. They found that a variety of STS interventions have been launched, including self-help programs, structured workshops, and in-person and online trainings. However, the field still lacked evidence that can be used to inform future STS assessments and interventions. While Sprang et al.’s study provided a thoughtful discussion of existing studies and future directions, their work remained a largely descriptive review rather than a systematic collection and analysis of evidence.

Despite the prior review efforts to date, calls to synthesize and evaluate empirical evidence on VT interventions have continued (e.g., Molnar et al., 2017). Moreover, there is a pressing need to summarize and assess extant VT interventions research both to take stock of the research conducted so far and to help move the field forward. For these reasons, we aimed to conduct a comprehensive scoping review of existing VT intervention research.

## Method

Considering that the existing literature regarding VT interventions was heterogeneous in nature, we adopted a scoping review approach, underpinned by Arksey and O’Malley’s (2005) strategy. A scoping review aims to map a body of literature in a field of interest. This approach addresses broad areas of evidence, exploring breadth rather than depth, and is useful when a topic is still in its early stages of development (Arksey & O’Malley, 2005). In order to strengthen rigor for this review method, Arksey and O’Malley developed a framework for conducting a scoping review, which includes five key phases: identifying the initial research questions, identifying relevant studies, study selection, charting the data, and collating, summarizing, and reporting the results (Arksey & O’Malley, 2005). Our review included these phases.

### Identifying the Research Questions

The focus of our review was the exploration of key aspects of VT interventions for service providers working with people who have experienced traumatic events. Specifically, we posed the following research questions to guide the search: (1) What is the extent, range, and nature of VT intervention studies for service providers? (2) What is known from this existing literature about the effectiveness of the VT interventions? (3) What are the implications for future practices and research based on the findings?

### Identifying Relevant Studies

Arksey and O’Malley (2005) suggested that a wide definition of key words for search terms should be adopted to obtain “broad coverage” of the available literature. Initial key concepts and search terms were developed to capture a broad range of literature regarding VT. Then, our research team consulted with a university reference librarian to refine search key words and appropriate literature databases based on our research questions. Using the recommended databases and search terms formats, beginning in October 2019 and ending in November 2019, we conducted the literature search through five electronic databases: PsycINFO, Social Service Abstract, PubMed, Cumulative Index to Nursing and Allied Health Literature, and Web of Science. We created four search terms categories, namely, VT terms, intervention terms, service provider terms, and client terms, and used combinations of the terms in the four categories (Table 1).

**Table 1.** Search Terms Categories and an Example of Search Terms Combinations.

Categories	Search Terms
Vicarious trauma terms	vicarious trauma*, secondary trauma*, secondary trauma* stress, compassion fatigue
Intervention terms	intervention, program, education, training, workshop, course, curriculum, approach, service, randomized controlled trial, experimental design
Service provider terms	social worker, counselor, health personnel, healthcare provider, doctor, nurse, physician, medical staff, hospital staff, service provider, therapist, clinician, victim advocate, mental healthcare provider, psychologist, substance abuse trainer
Client terms	trauma, violence, abuse, trafficking
An example search in PubMed database	((((Vicarious trauma[tw] OR secondary trauma[tw] OR secondary trauma stress [tw] OR vicarious posttraumatic growth [tw] OR compassion fatigue[mesh])) AND (Program evaluation[mesh] OR education[mesh] OR training OR workshop OR course OR curriculum OR intervention OR approach OR service OR program evaluation OR education OR Randomized Controlled Trial OR experimental design OR Evaluation studies)) AND (Social worker[mesh] OR counselor[mesh] OR health personnel[mesh] OR child Health Services/standards[mesh] healthcare provider OR doctor OR nurse OR physician OR medical staff OR hospital staff OR service provider OR therapist OR clinician OR victim advocate OR mental healthcare provider OR psychologist OR substance abuse trainer OR counselor OR social worker OR health personnel)) AND (Trauma[tw] OR violence[tw] OR abuse[tw] OR trafficking[tw])

### Study Selection

We examined the resulting articles based on the following inclusion criteria: (1) original research in peer-reviewed journals; (2) full texts available online with no exclusions with regard to publication dates; (3) human subjects; (4) study participants/sample includes professionals working with people experiencing traumatic events such as abuse and assault, grief and loss, human trafficking, military combat, natural disasters, and terrorism; (5) intervention-based studies focusing on VT, STS, and CF; and (6) peer-reviewed articles available in English. Thus, we excluded nonempirical articles, such as program manuals, books and book chapters, concept papers, and nonpeer review/gray literature reports on empirical studies (using qualitative, quantitative, or mixed methods), including unpublished research reports or dissertations.

**Figure 1.**
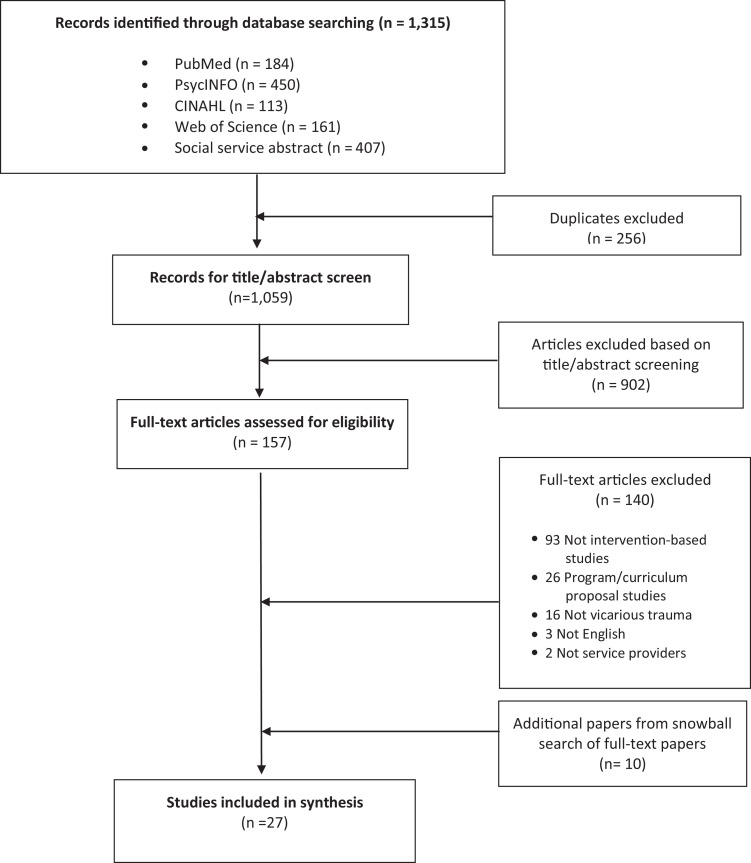
Preferred Reporting Items for Systematic Reviews and Meta-Analyses flow diagram of completed search.

Our search yielded 1,315 articles (see Figure 1). After the removal of duplicate articles (*n* = 256), we reviewed each article’s title and abstract based on the inclusion and exclusion criteria. The review produced a selection of 157 articles for full text review. Our team evaluated the articles in-depth by reading the full text and excluding articles (*n* = 140) if they did not cover VT, intervention research, or human subjects, if they were in the form of narratives, expert opinions, or review articles, or if they were not in English. Following the full-text review, a total of 17 studies were selected for the study. In addition, in an effort to be as comprehensive as possible in evidence identification, we conducted a backward search of the sources cited in each article as a means of identifying additional literature that might have been missed in our database searches. Our snowball search efforts added 10 articles meeting inclusion criteria for this review.

### Data Charting and Collating

To chart and collate our review of selected articles systematically, our research team developed a data abstraction tool. We designed this tool to obtain detailed information to the extent that such information was presented in each article, including information on VT interventions, study aims and method, study results, strengths, and limitations. Three members of our research team independently read full texts and then gathered detailed information using the abstraction tool. Before this process fully began, all team members read the same two articles and shared findings both to pilot the tool and to reach review consensus and consistency. After all abstractions were completed, one member of the research team independently reviewed each abstraction along with the article form to ensure accuracy and completeness across all reviews.

### Summarizing and Reporting the Results

The final stage of Arksey and O’Malley’s (2005) scoping review framework summarizes and reports findings. Table 2, Table 3, and Table 4 provides key information for each of the reviewed studies, noting the study characteristics, the types of VT interventions evaluated, and relevant findings.

**Table 2.** Vicarious Trauma Interventions—Study Characteristics, Relevant Findings, and Outcomes.

Author	Study Background Country Study Sample Service Delivery Setting	Intervention Name Theoretical Background Components Structures	Research Methods Type of Studies Design Data Collection	Key Outcomes and Measures ^a^	Key Study Findings ^b^
Psychoeducation					
Adimando (2018)	United States 24 Nurses Pediatric emergency department and department of psychiatry at a large urban hospital setting	No formal name—workshop to increase awareness of and prevent compassion fatigue (CF) No theory or model specified Self-care and stress management activities and discussion for individualizing self-care techniques In-person group sessions: One session, 1 hr	Mixed method Preexperimental (one group pre–posttest design) Cross-sectional (two time point)	Primary outcomes: CF (Compassion Fatigue Knowledge Test), quality of life (Professional Quality of Life Scale [ProQOL]) Qualitative interview questions: Open-ended questions asking workshop effectiveness, quality, and satisfaction	Increase in knowledge of CF Qualitative data indicated a generally positive response to the intervention
Allen et al. (2017)	Australia 25 Obstetrics/gynecology doctors A large metropolitan hospital	No formal name—Balint style discussion groups No theory or conceptual model identified All sessions facilitated by psychiatrists; participants discuss stories from their work (e.g., feeling of impotence) and stress management strategies In-person group sessions: Six monthly sessions, 1 hr	Quantitative Preexperimental (one group pre–posttest design) Longitudinal (three time points)	Primary outcomes: Compassion satisfaction, burnout and secondary traumatic stress (ProQOL-5)	Reduction in secondary traumatic stress and burnout Increase in compassion satisfaction
Berger & Gelkopf (2011)	Israel 90 Nurses A well-baby clinic at a hospital at Israel, in war (North) and terror (South) affected areas	No formal name provided, psychoeducational knowledge training No theory or conceptual model identified for providers’ stress management practice Provide screening tools for identifying children and parents at risk of developing stress-related problems; participants learn and practice self-maintenance tools In-person group sessions: 12 weekly sessions, 6 hr each	Quantitative Randomized control trial Cross-sectional (two time points)	Primary outcomes: Compassion satisfaction, burnout, and secondary traumatic stress (ProQOL-5) Secondary outcomes: Professional sense of self-efficacy, self-esteem, hope, and sense of mastery	1) Significant improvement on all outcome measures with the exception of self-esteem
Berger et al. (2016)	Australia 69 Educators At Linwood College, a coeducational secondary school in Linwood, a suburb of Christchurch, a town in Australia impacted by the 2011 earthquake	ERASE-Stress Intervention Adapted from the ERASE-Stress program (Berger, 2014) Discussion of 16 topics (e.g., mindfulness meditation [MM], body scan, and somatic pendulation, supporting grief and bereavement, boosting self-esteem**)** In-person group sessions in a 3-day intensive workshop (24 hr)	Quantitative Quasi-experimental Longitudinal (three time points)	Primary outcomes: Compassion satisfaction, burnout, and secondary traumatic stress (ProQOL-5), post-traumatic stress (Posttraumatic Check List) Secondary outcomes: Optimism, post-traumatic stress; coping, resilience, professional self-efficacy, and hope	Significant improvement on all outcome measures including primary and secondary outcomes
Cieslak et al. (2016)	Poland 168 Health and human service professionals (health care providers, psychotherapists, police officers, firefighters, etc.) Various health and human service settings	Self-efficacy/cognitive behavior therapy online intervention Social cognitive theory Cognitive-behavior treatment such as activity planning, skill training, and cognitive bias modification Individual online intervention through a designated website: Four sessions delivered over 4 weeks	Quantitative Randomized control trial Cross-sectional (two time points)	Primary outcomes: Secondary traumatic stress (Secondary Traumatic Stress Scale), secondary post-traumatic growth (Posttraumatic Growth Inventory) Secondary outcomes: Self-efficacy	Reduction in secondary traumatic stress Increase in secondary post-traumatic growth Increase in self-efficacy
deFouchier & Kedia (2018)	Central African Republic 98 National humanitarian aid workers Two international nongovernmental organizations acting with vulnerable children and their family in CAR: Children’s Villages and Action Contre la Faim	No formal name provided, stress management seminar Intervention was developed in accordance with the first phase of the CBT protocol for acute stress disorder and PTSD (1) psychoeducation on normal, chronic, and traumatic stress; (2) strengthening of physical, cognitive, behavioral, spiritual, and emotional coping strategies In-person group sessions: One session, 3 hr	Mixed method Nonexperimental Cross-sectional (two time points)	Primary outcomes: PTSD (Harvard Trauma Questionnaire), anxiety, and depression (Hopkins Symptoms Checklist) Qualitative interview questions: No details about qualitative instrument provided	Quantitative outcomes: significant reductions in PTSD, anxiety, and depression Qualitative outcomes: Benefits most appreciated were improved sleep and better ability to manage stress
Flarity et al. (2016)	United States 55 Forensic nurses Urban hospital ED, Level II trauma center	No formal name provided, educational seminar No theory or conceptual model specified Learned self-awareness of compassion fatigue and self-care activities In-person group seminar; one session, 4 hr	Quantitative Nonexperimental Cross-sectional (two time points)	Primary outcomes: Secondary trauma stress, compassion satisfaction, and burnout (ProQOL-5)	Increase in compassion satisfaction Reduction in burnout and secondary trauma stress
Flarity et al. (2013)	United States 73 Nurses Two emergency department at a hospital	Compassion fatigue resiliency No theory or conceptual model specified Learned self-awareness of CF and self-care activities In-person group seminar; one session, 4 hr	Mixed method Nonexperimental Cross-sectional (two time points)	Primary outcomes: Secondary trauma stress, compassion satisfaction, and burnout (ProQOL-5) Qualitative interview questions: No details about qualitative instrument provided	Increase in compassion satisfaction Reduction in burnout and secondary trauma stress Qualitative outcomes cited included ability to develop a self-help method
KleiVT, Secondary Trauma Stress, and Compassion Fatiguen et al. (2017)	United States 17 Health care professionals; nurses, physicians, and counselors/social workers Inpatient palliative care department and neonatal advanced practice providers at a medical center	A professional development program Biopsychosocial model Self-assessments and self-care strategies In-person, group-setting–based educational seminars: Three sessions, 90 min each, held 2 weeks apart	Quantitative Nonexperimental Longitudinal (three data collection points)	Primary outcomes: Secondary trauma stress, compassion satisfaction, and burnout (ProQOL-5)	Reduction in CF Small reduction in burnout Secondary trauma stress remained almost unchanged
Meadors & Lamson (2008)	United States 185 Health care providers; majority of participants were nurses (62.2%) A children’s hospital	CF: Addressing the biopsychosocial needs of professional caregivers No theoretical model was specified Education of biopsychosocial symptoms associated with secondary traumatic stress and practice techniques to manage stress, grief, and CF In-person, group-setting–based educational seminars: One session per participant, 4 hr	Quantitative Nonexperimental Cross-sectional (two time points)	Primary outcomes: CF (a scale formulated by investigators) Secondary outcomes: Perceived level of stress	Increase in knowledge of warning signs of CF Increase in perceived feeding of resources to manage multiple stressors, grief, and trauma at work and home
Tucker et al. (2017)	Canada Third year medical trainees/students (*n* = 59) Canadian undergraduate medical school	Burnout and CF program Seven essential elements of the program evaluation process (Haji et al., 2013) A self-reflection exercise to increase awareness of one’s own signs and symptoms of distress and a review of evidence for strategies aimed at increasing resilience and coping skills In-person group workshop: One workshop, length not specified	Mixed method Nonexperimental Longitudinal (three time points)	Primary outcomes: Secondary trauma stress, compassion satisfaction, and burnout (ProQOL-5) Qualitative interview questions: After workshop open-ended questions regarding workshop experience; at the end of the academic year: in-person focus groups interview regarding program participation	Reduction in compassion satisfaction Increase in burnout between beginning and end of third year Qualitative themes emerged: Recognizing symptoms of CF and learning to manage one’s own stress
Wahl et al. (2018)	United States 20 Nurses Hospital trauma center	Peer Support Network (PSN) pilot project Scott three-tiered interventional model Online module focused on the development of a PSN, resiliency workshop, and PSN implementation training Online module: 20 min, one module; in-person group workshop: 3 hr, one session	Quantitative Nonexperimental/ Cross-sectional (two time points)	Primary outcomes: Compassion satisfaction, burn out, and secondary traumatic stress (measured by two scales, ProQOL-5 & Compassion Practice Instrument [CPI]) Secondary outcomes: Self-care	Primary outcomes showed nonstatistical improvements with only the Compassion Satisfaction subscale (CPI) showing statistically significant increases Secondary measures show scores changing between two times, but, the change was not significant
Weidlich & Ugarriza (2015)	United States Military health care providers; civilian RNs and licensed practical nurses, military RNs and medics Military medical treatment facility	The Army’s Care Provider Support Program Siebert’s characteristics of highly resilient people as flexible, able to react to change and bounce back after adversity Education on CF and developing an individualized care plan Group training in-person: One session, typically 1–2 hr long	Quantitative Nonexperimental Cross-sectional (two time points)	Primary outcomes: Compassion satisfaction, burn out, and secondary traumatic stress (ProQOL-5) Secondary outcomes: Resiliency and ways of coping	Reduction in burnout No significant changes in other outcomes including other primary and secondary outcomes
Williamson (2019)	United States 44 Nurses Emergency department of a large urban, academic, Level 1 trauma center	Curriculum about alcohol use disorders (AUD) and CF Watson’s Theory of Human Caring Didactic information about CF and AUDs; case studies later presented for group discussion Online (for first six modules); group-based, in-person class (attended once); six online modules of 45 min each; one in-person class lasting 1 hr	Quantitative Nonexperimental Cross-sectional (two time point)	Primary outcomes: Compassion satisfaction, burn out, and secondary traumatic stress (ProQOL-5) Secondary outcomes: Attitudes toward alcohol use	No significant changes in CF and compassion satisfaction Positive change in attitudes toward alcohol use
Winblad et al. (2018)	United States 18 Health care providers: psychologists, social workers, medical doctors, psychiatrists, physical therapists, and other body-oriented therapists Diverse setting	Somatic experiencing Core Response Network Practice self-regulation skills, work in consultation groups, and have regular sessions that develop self-regulation capacity and increase skills as trauma-treating therapists In-person group sessions: Three beginning modules of four 6-hr days; three intermediate modules of four 6-hr days; two advanced modules of six 6-hr days	Quantitative Nonexperimental Longitudinal (four time points)	Primary outcomes: Quality of life (World Health Organization Quality of Life-Brief), psychological symptoms (Patient Health Questionnaire)	Increase in quality of life Reductions in anxiety and somatic symptoms
Xu et al. (2016)	China 579 Health care providers, physician, nurse, and other Hospitals	Positive Psychological Intervention Stress Management and Resiliency Training Psychological health education: (1) psychosomatic improvement, (2) psychosomatic self-rating, (3) emotion management, and (4) sharing growth In-person group sessions: Four courses, 30 min each	Quantitative Nonexperimental Cross-sectional (two time points)	Primary outcomes: Post-traumatic growth (post-traumatic growth inventory)	Increase in post-traumatic growth Subgroup analysis: Post-traumatic growth varied depending on gender, type of service position, and education level
Zajac et al. (2017)	United States 91 Health care providers, registered nurse, oncology care associate, and other Oncology/cancer center	No formal name: intervention to provide bereavement support to staff after patient deaths Morse’s (2001) praxis theory of suffering Debriefing sessions questions: How did you help the patient/family through this transition? What example of colleague collaboration was most noteworthy in this patient experience? What impact will this patient’s death have on you? In-person group sessions: occurred after each patient death and lasted 3 months; time of each session was noted but varied	Quantitative Nonexperimental Cross-sectional (two time points)	Primary outcomes: Compassion satisfaction, burn out, and secondary traumatic stress (ProQOL-5) Secondary outcomes: Patient satisfaction	Increase in compassion satisfaction No significant changes in burnout or secondary traumatic stress Patients’ perception of nurses’ skills improved significantly
Mindfulness					
Delaney (2018)	Scotland 18 Nurses Diverse department (Cancer Care, Cardiology, Maternity, Midwifery, Intensive Care, and Urology) at a hospital	Mindfulness Self-Compassion Training No theory or conceptual model was specified MM, loving kindness meditation, and compassion meditation In-person group sessions: one 2½-hr training session/week for 8 weeks, participation in a half-day retreat	Mixed method Nonexperimental Cross-sectional (two time points)	Primary outcomes: Compassion satisfaction, burn out, and secondary traumatic stress (ProQOL-5) Secondary outcomes: Mindfulness, self-compassion, and resilience Qualitative interview questions: Open-ended questions asking workshop effectiveness, quality, and satisfaction	Secondary trauma and burnout declined significantly; resilience, mindfulness, and compassion satisfaction scores increased Qualitative outcomes: multiple themes emerged—acceptance, mindful awareness, and less self-criticism
Dutton et al. (2017)	United States 18 Counselors, advocates, and lawyers who deliver services to survivors of child abuse, domestic violence, and sexual assault. The retreat was held near Ojai, CA	The Joyful Heart Foundation’s Holistic Healing Arts Retreat Humanistic or person-centered framework (a) A focus on the present, not past or future; (b) congruence of retreat practitioners: willingness to relate transparently to participants while maintaining appropriate boundaries; (c) empathy and a desire to understand participants’ perspectives; and (d) unconditional positive regard or acceptance without judgment of each participant 4-Day retreat	Mixed method Nonexperimental Longitudinal (four time points)	Primary outcomes: CF, compassion satisfaction, and burnout (ProQOL-5) Secondary outcomes: Somatic symptoms, insomnia, depressive symptoms, hope, self-efficacy, and self-Esteem Qualitative interview questions: Subjective thoughts and reactions throughout the retreat	Quantitative outcomes: positive changes in post-traumatic stress, perceived stress, fatigue, satisfaction with life, burnout, secondary traumatic stress, somatic symptoms, insomnia, and depressive symptoms Qualitative outcomes: Participants’ responses reflect greater attention to self-capacities
O’Mahony et al. (2016)	United States 13 Health care providers; chaplains (*n* = 5), nurses (*n* = 3), physicians (*n* = 3), and social workers (*n* = 2) Children’s hospitals with specialized end-of-life care services	Mindfulness training Framework of psychological flexibility theory Didactics to educate on topics of psychological flexibility, palliative care, and trauma and experiential mindfulness and communication exercises In-person group-based settings: Nine sessions (a half-day session on the first and last week and seven 2-hr sessions held weekly	Quantitative Nonexperimental Longitudinal (three time points)	Primary outcomes: Experiential avoidance (Acceptance and Action Questionnaire Version II), burnout (Maslach Burnout Inventory), depression (Beck Depression Inventory), cognitive fusion (Cognitive Fusion Questionnaire), and PTSD (PTSD Symptom Checklist)	Reduction in depression Reduction in PTSD Burnout, cognitive fusion, and experiential avoidance remained nearly unchanged
Slatyer et al. (2017)	Australia 16 Nurses; full time (*n* = 12) and part time (*n* = 4) A hospital	Brief mindful self-care and resiliency (MSCR) program Theoretical model put forth by Rees et al. (2015) Introduction to mindfulness, weekly mindfulness skills seminars In-person, group-based workshop followed by weekly mindfulness seminars: One workshop session of 12 hr, four seminars of 1.5 hr	Qualitative Nonexperimental Cross-sectional (one time point)	Qualitative interview questions: Broad questions asking the participant to describe the strengths and weaknesses of the program	Five themes emerged that described the impact of the program: Gaining perspective and insight; developing feelings of inner calm; and talking time to care for self, perceived feasibility, and acceptability of the MSCR program
Wylde et al. (2017)	United States 95 Health care providers (Novice nurses participating in a pediatric residency program) A children’s hospital	Traditionally Delivered Mindfulness (TDM) and Smartphone Delivered Mindfulness (SDM): TDM: Adapted from a 2006 study by Mackenzie, Poulin, and Seidman-Carlson SDM: Philosophy of the Karma Kagyu lineage of Tibetan Buddhism TDM: Walking, standing, sitting, yogic postures, lying down, and eating SDM: Headspace meditation techniques TDM: Group-based, in-person sessions: One session per week for 4 weeks SDM: Individual use of the Headspace application: One app use per week for 4 weeks	Quantitative Quasi-experimental Cross-sectional (two time points)	Primary outcomes: Compassion satisfaction, CF, and burnout (Compassion Fatigue Self-Test) and trauma symptoms (PTSD Checklist—Civilian Version) Secondary outcomes: Mindfulness	SMD group showed marginally more compassion satisfaction and less burnout than TMD group SDM group had lower risk of CF than TDM group SDM group had higher level of mindfulness skills than TDM group
Art and recreational programs					
Boone & Castillo (2008)	United States 55 Domestic violence counselors It appears that participants read and wrote about the poems on their own and not in a centralized setting	Poetry therapy Pennebaker’s (1997) basic expressive writing paradigm Individuals in the “poetry group” were assigned poems to read and then wrote personal responses to the poems Not clear	Quantitative Quasi-experimental, Cross-sectional (two time points)	Primary outcomes: Secondary PTSD (Impact of Events Scale)	Reduction in secondary PTSD
Hatzipapas et al. (2017)	South Africa 7 Community care workers serving HIV/AIDS-infected and -affected clients Two local community centers	Aerobic Laughter Therapy (ALT) ALT is a cognitive behavioral technique defined by the Association for Applied and Therapeutic Humor Physical warmup activities, breathing techniques, and laughter exercises that combine acting and playful visualization techniques (Kataria, 2002). Daily 10- to 15-min group laughter sessions for a month	Qualitative focused mixed method Quasi-experimental Cross-sectional (two time point)	Qualitative interview questions: Examples of questions were: (1) How do you experience your work as a care worker? (2) How do you know when you are stressed? (3) How does stress affect your work with the children in your care? Quantitative outcomes: Perceived stress (Perceived Stress Scale) and anxiety and depression (Hospital Anxiety and Depression Scale)	Qualitative outcomes reported by participants included more positive emotions, positive coping, improved interpersonal relationships, and improvement in care work Quantitative outcomes: positive changes in stress, anxiety, and depression
Newman et al. (2015)	South Africa 17 Health care providers (child psychiatrists, doctors, nurses, and other providers) Child and adolescent mental health unit at a psychiatric hospital	Drumming group Built on ancient history of drumming, which has been used in cultural healing practices Each session consists of teaching basic hand techniques and rhythms and using the drums as a means of expression Held Monday, Wednesday, Friday for 30 min before work	Qualitative Nonexperimental Cross-sectional (one time point)	Qualitative interview questions: Six open-ended questions (e.g., How does drumming make you feel? Does drumming affect your work in any way? If so, how?, Does drumming impact your relationships with other members of the group?)	Qualitative themes identified as described the impact of the program: Sense of belonging, relaxation, energy and productivity, learning, mood, etc.
Alternative medicine programs					
Buchanan et al. (2018)	United States 42 Health care providers, registered nurses (*n* = 29), physical therapists (*n* = 2), occupational therapists (*n* = 1), pharmacists (*n* = 1), and others (*n* = 9) Cardiovascular division of a hospital	Auricular acupuncture National Acupuncture Detoxification Protocol or NADA Protocol Placing the needles on the five specific points on the external ear to release of negative emotions associated with specific organs In-person individual sessions: 1/week for 30 min, conducted in a quiet and peaceful room	Quantitative Nonexperimental Cross-sectional (two time points)	Primary outcomes: Anxiety (State-Trait Anxiety Inventory), workplace engagement (Utrecht Work Engagement Scale)	Reduction in State and trait anxiety Increase in scores on work engagement
Novoa & Cain (2014)	United States 67 Service providers; social work professionals (*n* = 34), student interns (*n* = 28), and counselors (*n* = 3) Not specified; variety of settings where mental health professionals can work	Reiki treatment (an energy therapy modality) No theoretical model was specified Sessions started with the participant lying on their back. The practitioner started at the head and worked toward the feet, keeping hands approximately 1.5–2.0 in. away from the body One-on-one, in-person sessions with Reiki practitioner: Four sessions every week, 50-min sessions	Quantitative Randomized Cross-sectional (two time points)	Primary outcomes: Compassion satisfaction and secondary traumatic stress (ProQOL-5) Secondary outcomes: Depression, anxiety, anger, and hope/hopelessness	No significant differences were found between treatment group, placebo, and control group in any outcomes

*Note.* PTSD = post-traumatic stress disorder; ASD = acute stress disorder; CBT = cognitive behavioral therapy; CF = compassion fatigue; RN = registered nurse; LPN = licensed practical nurse; CS = compassion satisfaction.

^a^ Only scales for the primary outcomes are indicated. ^b^ Improvements of outcome measures in the section of key study findings indicate that the changes are statistically significant with the *p* values less than .05.

**Table 3.** Vicarious Trauma Intervention Research Strengths and Limitations.

Author	1) Strengths 2) Limitations
Psychoeducation	
Adimando (2018)	Short and feasible intervention, validated measures, and mixed methods Small sample size, no control/comparison group, only two data collection points, and lack of detail on qualitative analysis
Allen et al. (2017)	In-depth discussion program facilitated by psychiatrists, three data collection points, and validated measures Small sample size, no control/comparison group, and little detail on the intervention program
Berger & Gelkopf (2011)	Detailed curriculum, validated measures, control/comparison group (wait-list), rigorous analysis strategies, and relatively large sample size Intensive program sessions may be less feasible in certain types of settings and only two data collection points
Berger et al. (2016)	Adapted training to address the needs of diverse participants, given the ethnic/cultural backgrounds, control/comparison group, relatively large sample size, and three data collection points Intensive program sessions may be less feasible in certain types of settings and the measures of optimism and personal self-efficacy were based on single items
Cieslak et al. (2016)	Strongly evidence-based intervention model (targeting self-efficacy as well as using cognitive therapy), control/comparison group, relatively large sample size, control/comparison group, and rigorous analysis strategies Selection bias (online participation), high attrition, large dropout rate, and only two data collection points
deFouchier & Kedia (2018)	CBT-based feasible and cost-effective intervention, clinical psychologists administered the intervention, relatively large sample size, and mixed methods No control/comparison group, no description of qualitative methods used, and only two data collection points
Flarity et al. (2016)	Brief, novel intervention and validated measures Small sample size, no control/comparison group, and only two data collection points
Flarity et al. (2013)	Short and feasible intervention, mixed method, relatively large sample size, and validated measures Only two data collection points, no control/comparison group, and high dropout rate
Klein et al. (2017)	Biopsychosocial model-based intervention, three data collection points, and validated measures Small sample size and no control/comparison group
Meadors & Lamson (2008)	Short and feasible intervention and large sample size No control/comparison group, only two data collection points, little detail on the intervention program, and short follow-up
Tucker et al. (2017)	Short and feasible intervention, mixed methods, three data collection points, and validated measures Small sample size and high attrition
Wahl et al. (2018)	Scott three-tiered interventional model-based intervention and validated measures Small sample size, no control/comparison group, and only two data collection points
Weidlich & Ugarriza (2015)	Short and feasible intervention and validated measures Small sample size, only two data collection points, short follow-up, and high dropout rate
Williamson (2019)	Control/comparison group and validated measures Small sample size, only two data collection points, and difficult to determine engagement and understanding of participants via online modules
Winblad et al. (2018)	Time-intensive trainings deeply exploring resilience and secondary trauma, four data collection points, and validated measures Small sample, no control/comparison group, and high dropout rate
Xu et al. (2016)	Incorporated context-specific elements (i.e., Chinese traditional cultural ideals) into the program, large sample size, and validated measures No control/comparison group and only two data collection points
Zajac et al. (2017)	Theory-driven program, validated measures, and moderately sized sample No control/comparison group and only two data collection points
Mindfulness	
Delaney (2018)	Manualized intervention, mixed methods, validated measures/scales, and rigorous analysis strategies Small sample size, high attrition, only two data collection points, and no control/comparison group
Dutton et al. (2017)	Very detailed intervention description, mixed methods, four data collection points, validated measures, and rigorous analysis strategies No control/comparison group, small sample size, and participants had 5 days off work to attend the retreat, which may be infeasible or too costly for some settings
O’Mahony et al. (2016)	Theory-driven program, validated measures, and three data collection points Small sample size and no control/comparison group
Slatyer et al. (2017)	Theory-driven program and intensive intervention sessions, which facilitated program delivery Lack of detailed information regarding qualitative analysis techniques to ensure trustworthiness of the study findings
Wylde et al. (2017)	Control/comparison group, strong statistical analyses, and validated measures Lack of detailed information regarding the intervention and only two data collection points
Art and recreational programs	
Boone & Castillo (2008)	A novel and unique intervention, control/comparison group, and validated measures Not structured intervention, recruitment methods may have led to bias, small sample size, and only two data collection points
Hatzipapas et al. (2017)	A novel and unique intervention and mixed-methods study Lack of detailed information on the intervention, small sample size, no control/comparison group, and only two data collection
Newman et al. (2015)	A novel and unique intervention, dual coding of qualitative data, and detailed explanation of data analysis Relatively low response rate (17 of 30 individuals)
Alternative medicine programs	
Buchanan et al. (2018)	A novel and unique intervention, detailed description of recruitment methods, and validated measure Small sample size, no control or comparison group, and only two data collection points
Novoa & Cain (2014)	Experimental placebo-control design with random assignment to one of the three treatment groups (control, placebo, or Reiki treatment), validated psychological instruments, and intervention description well detailed Small sample size and only two data collection points

*Note.* CBT = cognitive behavioral therapy.

**Table 4.** Summary of Critical Findings.

Vicarious trauma (VT) interventions mostly targeted health care professionals and were delivered as the following types of programs, psychoeducation, mindfulness interventions, art and recreational programs, and alternative medicine therapies
VT interventions were generally self-care based and tended to focus on general stress reduction and health promotion rather than addressing the specific effects of VT
The VT interventions generally showed positive effect in their key outcomes. In particular, interventions delivered over the longer term in a group setting showed the most promise in addressing service providers’ VT symptoms. However, future systematic evaluations are needed to determine the efficacy of such interventions, as well as investigate the efficacy of different intervention approaches

## Results

### Study Characteristics

This scoping review included 27 empirical studies, which were diverse in their samples, methods, intervention programs, and findings.

#### Study setting and participants

The 27 articles identified were published between 2008 and 2019, with the majority published within the past 6 years (*k* = 23, 85%). More than half of the studies were conducted in the United States (*k* = 16, 59%), whereas the rest of the studies were conducted in other countries (*k* = 11, 41%; e.g., Australia, South Africa, Canada, Poland, Scotland, China, Israel, and Central African Republic). The majority of the studies (*k* = 19, 70%) investigated the efficacy of VT interventions for health care providers working in diverse settings, including palliative care, cancer care, obstetrics/gynecology, and hospital emergency and trauma centers. Meanwhile, the other eight studies (*k* = 8, 30%) were targeted to human service professionals who respond to clients’ violence and trauma issues in a variety of settings, including counselors, lawyers, social workers, and teachers. Of the documents reviewed, three studies examined VT interventions for novice professionals, such as nurses entering the field (Wylde et al., 2017), medical student interns (Tucker et al., 2017), and mental health interns (Novoa & Cain, 2014). In addition, of the total literature, three studies sampled professionals experiencing dual trauma in war, terror, and natural disaster affected areas (Berger et al., 2016; Berger & Gelkopf, 2011; Weidlich & Ugarriza, 2015). In these situations, service providers may suffer symptoms of both primary trauma (as a result of the war, terror, or disaster itself) and secondary trauma (as a result of working with traumatized clients and patients).

#### Research method

The majority of the empirical studies (*k* = 18, 66%) were quantitatively focused research; only two studies (7%) used qualitative methods, namely, in-depth interviews and focus group interviews (Newman et al., 2015; Slatyer et al., 2017). Seven articles (25%) adopted mixed method designs that included collecting quantitative evaluation data and qualitative interviews. With the exception of Hatzipapas et al.'s (2017) study, which adopted a qualitatively focused mixed design, the other six studies used a quantitatively focused mixed method design. For articles using a quantitative approach, most of the studies (*k* = 14, 77%) adopted preexperimental designs, which is a one-group pre- and posttest design without a control or comparison group. Only four studies (14%) adopted a randomized control trial (RCT) design, which assigned control and intervention groups through random assignment (Berger & Gelkopf, 2011; Boone & Castillo, 2008; Cieslak et al., 2016; Novoa & Cain, 2014). Of the four RCT studies, two studies used an RCT design using an online format but did not provide specific information for their RCT process (Boone & Castillo, 2008; Cieslak et al., 2016). Another two studies (7%) used quasi-experimental designs with a comparison group but not random assignment (Berger et al., 2016; Wylde et al., 2017). Overall, apart from the two qualitative studies, most studies adopted simple pretest–posttest group design with two data collection points (*k* = 18, 66%). Only seven studies adopted longitudinal design with three or more data collection points (*k* = 7, 25%).

#### Major measurements

Of the 25 quantitative and mixed-method studies, 21 studies (77%) collected data from participants concerning VT-relevant constructs, such as STS, CF, compassion satisfaction, post-traumatic stress, and secondary posttraumatic growth, as their primary outcomes. The Professional Quality of Life Scale (ProQOL) Version 5 (Stamm, 2010) was the most commonly used scale for measuring VT-relevant constructs (*k* = 14, 51%). Using Figley’s (1995) definition of CF and its constructs, Stamm (2010) developed the ProQOL-5 scale (Wells-English et al., 2019). The 30-item ProQOL-5 includes three subscales: Compassion Satisfaction, Burnout, and Secondary Trauma Stress where respondents indicate how frequently they have experienced each in the past 30 days (Stamm, 2010). Consistent with this, Wylde’s (2017) study used the Compassion Fatigue Self-Test (Figley & Stamm, 1996) which is a previous version of the ProQOL-5. Compassion Fatigue Self-Test is a 66-item test measuring individuals’ levels of compassion satisfaction, CF, and burnout in their role as a helper. The other two studies measured VT-relevant outcomes by the Secondary Traumatic Stress Scale (STSS) which contains three 17-item subscales (Intrusion, Avoidance, and Arousal). The STSS assessed the frequency of STS symptoms experienced by service providers in the past 7 days using a 5-point, Likert-type response format, congruent with the DSM-IV-TR definition of PTSD (Bride et al., 2004). These three instruments, ProQOL-5, Compassion Fatigue Self-Test, and STSS, have demonstrated strong internal reliability in previous studies (Bride et al., 2004; Figley & Stamm, 1996; Stamm, 2010). Notably, the other four studies (14%) measured the efficacy of VT interventions through general mental health outcomes such as general PTSD, perceived stress, avoidance, anxiety, depression, and workplace engagement (Buchanan et al., 2018; de Fouchier & Kedia, 2018; Hatzipapas et al., 2017; O’Mahony et al., 2016). Aside from primary outcomes, nine studies used additional secondary measures (e.g., professional sense of self-efficacy, self-esteem, hope, a sense of mastery, perceived level of stress, self-care, resiliency, ways of coping, and attitudes toward alcohol use) to examine the effectiveness of VT interventions (Berger et al., 2016; Berger & Gelkopf, 2011; Cieslak et al, 2016; Meadors & Lamson, 2008; Wahl et al., 2018; Weidlich & Ugarriza, 2015; Williamson, 2019; Xu et al., 2016; Zajac et al., 2017). With respect to the measurement of qualitative studies, interview questions mainly consisted of brief feedback on the intervention (e.g., overall subjective thoughts about the intervention). For example, the interview asked broad questions on the strengths and weaknesses of the program (Slatyer et al., 2017) and how the program made participants feel or how it affected their work (Newman et al., 2015).

### Key Findings

In the reviewed studies, the majority of the VT interventions focused on psychoeducation (*k* = 17, 62%) and mindfulness (*k* = 5, 18%) approaches, with overlap in their key activities. The other program types include art and recreational programs, such as a drumming group, aerobic laughter therapy, and poetry therapy (*k* = 3, 11%) and complementary and alternative medicine, such as auricular acupuncture and energy therapy (*k* = 2, 7%). Comprehensive details are provided regarding key program activities, delivery methods, effectiveness, and limitations.

### Psychoeducation

#### Key activities and delivery methods

Seventeen studies implemented and evaluated psychoeducational-focused interventions. Generally, the psychoeducational approach included (1) education on the early symptoms of VT, how to be cognizant of VT, and physical and psychological signs and symptoms of VT; (2) education on self-care and stress management activities and techniques (e.g., mindfulness practices); and (3) development and application of individual self-care plans. However, the findings also showed considerable diversity across all these interventions’ theoretical underpinnings and program activities. For example, some VT interventions used evidence-based psychological models such as cognitive behavior therapy (e.g., Cielak et al., 2016; de Fouchier & Kedia, 2018). Alternatively, some interventions focused on brief, professional stress management seminars with education on self-care activities. Notably and in addition, some psychoeducational interventions included additional components or methods including professional skills trainings (Berger & Gelkopf, 2011; Williamson, 2019) and peer support networks (Wahl et al., 2018).

With respect to delivery methods, psychoeducational-focused programs tended to consist of in-person group sessions, including didactic presentations and small activities. However, the length and intensity of the interventions ranged from 1 to 12 sessions and from 1 to 6 hr each session meeting. Specifically, about half of the psychoeducational programs (*k* = 8) provided a short program through a one-time session rather than programs with multiple sessions. Notably, some interventions were implemented through unique formats, such as an intensive 3-day workshop (Berger et al., 2016), psychiatrist-led Balint-style discussion groups (Allen et al., 2017), an online format (Cieslak et al., 2016; Williamson, 2019), and hybrid formats, which included both in-person and online meetings (Wahl et al., 2018).

#### Effectiveness

The majority of the psychoeducational programs (*k* = 13, 76%) showed promise with regard to key outcomes, which include VT, STS, CF, burnout, and other negative psychological symptoms, including anxiety, as measured with standard scales, especially the ProQOL-5. However, four studies showed mixed findings across multiple outcome measures and in follow-up (Klein et al., 2017; Weidlich & Ugarriza, 2015; Williamson, 2019; Zajac et al., 2017). As an example, a resilience intervention for health care workers showed a positive change in compassion satisfaction and a small reduction in burnout, but no change in STS during pre- and post-follow-up (Klein et al., 2017). Even though some studies were conducted using strong research designs, including as examples, large sample sizes, longitudinal designs, and randomization (e.g., Berger et al., 2016; Cieslak et al, 2016), most of the psychoeducational studies (*k* = 10, 37%) used one-group, pre- and posttest design, without control or comparison groups.

### Mindfulness

#### Key activities and delivery methods

Five studies adopted mindfulness interventions to prevent and reduce service providers’ experience of VT. Even though there were variations in major program components and delivery methods, these interventions generally covered an introduction to mindfulness and mindfulness practices such as meditation, yoga, and body movement. However, some mindfulness interventions also included psychoeducation (Dutton et al., 2017; Slatyer et al., 2017). For example, a brief mindful self-care and resiliency program provided a 1-day psychoeducational workshop on CF, resiliency, and mindfulness, followed by four weekly mindfulness skill seminars (Slatyer et al., 2017). In terms of delivery methods, mindfulness interventions generally consisted of in-person group sessions, except for one smartphone-based mindfulness training (Wylde et al., 2017). These interventions delivered content through a combination of mindfulness seminars and practices and generally seemed to have more or lengthier sessions compared to psychoeducational programs. In particular, one study implemented a 4-day retreat for violence advocates to help participants find balance and heal their minds, bodies, and spirits based on a holistic approach (Dutton et al., 2017).

#### Effectiveness

A review of all the mindfulness interventions demonstrated their effects, not only on VT-related symptoms (e.g., STS, burnout, and PTSD) but also on outcomes related to mind–body wellness (e.g., somatic symptoms, insomnia, and mindfulness). However, while a retreat-based intervention showed positive effects of reduced VT, which lasted until the 3-month follow-up, the effects were no longer seen by the 6-month follow-up (Dutton et al., 2017). Meanwhile, a smartphone-based mindfulness training showed similar or better effects of reduced VT compared to traditional, in-person training format (Wylde et al., 2017). Overall, mindfulness interventions study used a single group pre- and postdesign, except for one study with a quasi-experimental design (Wylde et al., 2017). In qualitative findings, participants commonly reported greater attention to self-acceptance and mindful awareness and less self-criticism through mindfulness (Dutton et al., 2017; Slatyer et al., 2017). Even though qualitative studies provided a brief explanation of the data analysis process, the authors lacked an explanation of the steps they took to ensure trustworthiness (e.g., triangulation, peer debriefing, and audit trail; Lietz & Zayas, 2010).

### Art and Recreational Programs

#### Key activities and delivery methods

Three art and recreational programs (online poetry therapy, aerobic laughter therapy, and a drumming group program) were implemented to decrease professionals’ trauma-related symptoms (Boone & Castillo, 2008; Hatzipapas et al., 2017; Newman et al., 2015). Overall, these interventions adopted novel program approaches that were empirically supported in previous studies. However, the interventions seemed to be unstructured in that the descriptions of the intervention programs lacked a detailed explanation. For instance, the poetry therapy designed for domestic violence counselors consisted of reading poetry provided by researchers and then writing emotionally expressive pieces that allowed the counselors to address and process their secondary PTSD through reflection on the poetry (Boone & Castillo, 2008). This program was implemented across three sessions, using online platforms; however, specific information on program delivery was not provided. Aerobic laughter therapy for community care workers consisted of physical warm-up activities, breathing techniques, and laughter exercises and was implemented 10–15 min a day for a month (Hatzipapas et al., 2017). The drumming group program, designed for health care professionals at a psychiatric hospital, was delivered by a psychologist who had experience in drumming (Newman et al., 2015). This program was held 3 times per week for 30 min before work for 18 months. Each session consisted of teaching drumming techniques and playing together as a means of expression.

#### Effectiveness

Domestic violence advocates who participated in the online poetry therapy with the online RCT study showed a decrease in secondary PTSD compared to a control group (Boone & Castillo, 2008). The study of the aerobic laughter therapy adopted a qualitative-focused, mixed method design (Hatzipapas et al., 2017). Qualitative findings showed that participants reported more positive emotions, positive coping, improved interpersonal relationships, and improvement in their work after exposure to laughter therapy. Quantitative findings showed that this laughter therapy was effective on reducing participant stress, anxiety, and depression. The drumming group for health care providers also used a qualitative research approach and showed that participants experienced a sense of belonging, relaxation, emotional expression, and an escape from VT (Newman et al., 2015). Even though two qualitative studies provided detailed data analysis techniques to assure research quality, they appeared to be largely based on exploratory research.

### Alternative Medicine Programs

#### Key activities and delivery methods

The reviewed studies included two alternative medicine programs (Buchanan et al., 2018; Novoa & Cain, 2014). An auricular acupuncture program was implemented for health care providers in the cardiovascular division of a hospital (Buchanan et al., 2018). In this therapy, an acupuncturist placed needles in five specific points on the participants’ external ear in order to facilitate the release of negative emotions associated with specific organs (e.g., the liver with anger, the lungs with sadness, and the kidney with fear). This therapy was implemented 5 times for 30 min, over the course of 16 weeks. Another alternative medicine program was an energy therapy called Reiki, which was delivered to different mental health service providers (Novoa & Cain, 2014). In this therapy, a Reiki practitioner started at the head and worked toward the feet, keeping their hands approximately 1–2 in. away from the participant’s body. This therapy aimed to heal using a holistic approach that incorporated mind, body, and spirit. A Reiki practitioner implemented four one-on-one, in-person sessions every week for 4 weeks.

#### Effectiveness

Auricular acupuncture therapy with a one-group, pre- and posttest design showed a significant reduction in participant anxiety and an increase in their overall work engagement scores compared to scores at baseline (Buchanan et al., 2018). However, this study did not measure vicarious trauma-related outcomes, even though the program aimed to cover CF. The Reiki treatment for mental health professionals adopted an RCT design and showed no significant difference in compassion satisfaction, STS, or burnout between the treatment and control groups (Novoa & Cain, 2014).

## Discussion

Given the critical role that VT plays in service providers’ mental health and well-being, as well as in the quality of service provision, it is important to understand the existing evidence on interventions used to address service providers’ VT. Through this current study, we aimed to investigate and examine the existing interventions designed to address service providers’ VT-related symptoms. Specifically, we sought to determine (a) the scope of currently available published literature on addressing service providers’ VT related to symptoms, (b) the effectiveness of VT interventions, and (c) the implications for future practice and research.

### Scope of VT Intervention Research

As previously mentioned, we identified 27 articles, published between 2008 and 2019, which evaluated interventions created to address health and human service providers’ VT-related symptoms, including STS and CF. The majority of the literature on VT interventions was conducted in the United States and adopted quantitative research or quantitatively focused mixed method designs. Generally, the studies reviewed adopted a single-group research design, without a control or comparison group with only four using RCT designs. VT-related outcomes were largely measured using a ProQOL-5 Scale, which includes CF, burnout, and secondary trauma stress. Literature on VT interventions mostly targeted health care professionals, while the rest of the literature targeted human service providers serving clients exposed to violence, abuse, and disaster. Although VT interventions that have been empirically investigated to date are heterogeneous and multidimensional in their approaches, the literature can be broadly divided into four categories: psychoeducation, mindfulness intervention, art and recreational programs, and alternative medicine therapy. We also note here that the review findings show that the key activities of the first two categories—psychoeducation and mindfulness interventions—often overlapped. Accordingly, the approaches used for VT interventions may not always be distinct in either practice or research.

### Effectiveness of VT Interventions

Due to the variations of study population, outcome measures, and research designs, assessing and establishing the promise of the various VT interventions determined in this review were not possible. Accordingly, and consistent with the scoping review approach taken for this study, this discussion largely reflects summaries of intervention outcomes but also delves into the details of statistical findings when possible.

Overall, findings showed that, regardless of intervention type, the VT interventions reviewed generally showed promising findings not only for primary outcomes related to VT symptoms but also in secondary outcomes (e.g., self-efficacy, job satisfaction, and mindfulness). In particular, the qualitative studies reviewed here showed that participants commonly reported more mindful awareness, less self-criticism, greater relaxation, an increased sense of belonging, and an escape from VT. However, the review findings also showed some mixed outcomes, with VT interventions being effective in decreasing some VT-related symptoms, but not effective in other outcomes (Klein et al., 2017). Such results indicate the multidimensional nature of VT-related symptoms and suggest that VT-related symptoms may need to be addressed using a tailored approach to each aspect of VT responses. In addition, the review findings tended to show that interventions delivered over the longer term in a group setting may help address service providers’ VT symptoms through peer support and comprehensive program approaches that address the complex nature of VT.

Despite the overall positive trends in the studies’ outcomes, we also need to consider the studies’ research design and limitations (Bercier & Maynard, 2015; Sprang et al., 2019). Generally, the studies reviewed adopted a single-group research design, without a control or comparison group, used small samples, and limited short-term follow-up. Concerning the studies’ follow-up periods, given that VT is multidimensional and presents complex symptoms, prior research has strongly recommended that VT needs to be measured through long-term follow-up (O’Mahony et al., 2016). In addition, only a few studies measured participants’ baseline trauma symptoms or previous trauma history (e.g., Wylde et al., 2017). Given that service providers’ personal trauma histories are critical risk factors on VT symptoms, studies that did not consider previous trauma might fail to control for confounding effects on the study outcomes. In the case of the qualitative research, studies often lacked information regarding how robust research methods were used to ensure study trustworthiness. To sum, while the investigated VT intervention research shows promise, the inconsistency and lack of rigor across this body of research means that little can be confidently said about the effectiveness of any approach to prevent VT, particularly when the rigor of these studies is paired with the heterogeneous nature of the intervention approaches.

### Implication for Practice and Policy

This scoping review demonstrates that existing VT interventions are generally self-care based and focus on stress management rather than addressing the specific symptoms of VT depending on service setting and type of trauma. Therefore, we call for an increase in efforts to tailor VT interventions to different service settings and participant characteristics, as well as greater attention to developing primary VT interventions at the organizational level. We detail specific recommendations in the following paragraphs.

First, we found that existing VT interventions, in particular psychoeducation and mindfulness interventions, generally focused on generic wellness and job stress reduction and used a similar format of key activities, such as increasing self-care, self-awareness, and mind–body skills. Given that VT-related symptoms are multidimensional, VT interventions should focus on trauma-related alterations in emotions, beliefs, and behaviors as well as on occupational skill development. Notably, VT interventions need to address specific effects of VT symptoms depending on service setting (e.g., health care workers are exposed to grief and loss, while those in a violence service setting are exposed to violence and abuse), type of trauma (e.g., indirect or dual, frequency, duration, and severity), and service providers’ individual characteristics. Thus, practitioners need to provide programs tailored to particular service settings, taking into account likely sources, types of trauma, and participants’ individual characteristics and backgrounds (Nuttman-Shwartz, 2015).

We also found that existing VT interventions seem to lack attention to potential protective and risk factors for VT. Prior studies show that VT-related symptoms and levels differ depending on individual characteristics including gender and age (Baum, 2016; Benuto et al., 2018), exposure to previous trauma (Rauvola et al., 2019) as well as work experience (Benuto et al., 2018). A recent systematic review regarding VT among child welfare and child protection professionals showed that personal trauma history was the most consistent risk factor for vicarious traumatization (Molnar et al., 2020). Thus, we recommend that future interventions should consider and attend to key factors such as participants’ age, race, gender, previous trauma history, and work experience in order to provide participants with focused, meaningful interventions that are tailored to their backgrounds, circumstances, and needs.

In addition, VT intervention programs must be designed with clear program targets and goals, defining whether they seek to offer preventative intervention or ameliorative treatment. With the exception of a few studies targeting students and interns (Tucker et al., 2017; Wylde et al., 2017), it was unclear in our review whether programs aimed to prevent potential VT symptoms or decrease already existing VT symptoms. Sprang and colleagues suggested that universal and primary preventative VT psychoeducation can be considered a prevention intervention, which aims to reduce risk factors and strengthen protective factors for all staff (Sprang et al., 2019). On the other hand, VT intervention can be delivered as treatment intervention which consists of selective, secondary intervention and clinical treatment for staff and who are experiencing STS (Sprang et al., 2019). Such interventions may helpfully include motivational interviewing and cognitive therapy which focused on clinical approach of service providers’ VT symptoms (Sprang et al., 2019). For all these reasons, designing frameworks for VT interventions which consider target group’s characteristics should precede the development and implementation of VT interventions.

We also found that the VT interventions reviewed were mostly based on individual-based self-care approach. Even though individual self-care appears essential to decrease VT-related symptoms, primary prevention on an organizational level may also be necessary to structurally and contextually lower the risk of potential VT among providers (Sprang et al., 2019). Theories of work stress as well as empirical studies have suggested that organizational culture, support, and resources may determine how service providers understand and respond to their VT-related symptoms (Molnar et al., 2017; Rauvola et al., 2019; Sprang et al., 2019). Accordingly, VT prevention activities should be implemented both at the organizational and individual levels. Suggestions for creating a VT-informed organizational culture might include increasing trauma-specific supervision, developing peer support networks, and providing opportunities for clinicians to receive mental health services and online support (Branson, 2019; Elwood et al., 2011). For example, VT-informed organizational readiness guides (Hallinan et al., 2019), STS-informed organizational assessments (Sprang et al., 2019), and training in STS core competencies in trauma-informed supervision (National Child Traumatic Stress Network, 2018) can be useful tools to assess and diagnose organizational capacity and readiness to address VT and can support organizations in providing VT-informed supervision to professionals at risk of developing VT (Sprang et al., 2019).

Despite the limitations of the research, the studies we reviewed suggest potentially promising approaches that can inform practitioners who are considering implementing VT interventions. In particular, the review findings suggest that interventions delivered over the longer term in a group setting may help address service providers’ VT symptoms. Behavioral and cognitive theories of PTSD treatment posit that trauma symptoms can be decreased through consistent emotional adjustment to traumatic experiences (O’Mahony et al., 2016). Refresher courses or periodic monitoring may increase awareness and processing of work-related traumas, which in turn may lead to inhabituation and an eventual improvement in well-being (Klein et al., 2017; O’Mahony et al., 2016). In addition, peer-group-based interventions can contribute to a sense of group cohesion and support, which can contribute to the prevention and mitigation of secondary traumatization symptoms (Berger & Gelkopf, 2011; Newman et al., 2015).

Lastly, policymakers and funders should encourage the development and evaluation of evidence-based programs and protocols designed to address VT among service providers. Although this review showed that there are potentially promising interventions for addressing VT, the field continues to need the development and evaluation of tailored programs. Moreover, such programs, once developed, should be rigorously studied for efficacy. In particular, we urge funders to support sufficient resources for innovative intervention development and rigorous evaluation, which includes funding support for randomized designs, large samples, and follow-up data collection.

### Research Implications

Future studies need to clearly define VT-related symptoms and assess the efficacy of interventions, using appropriate measurement for those specific VT-related symptoms. Across the articles we reviewed, there seemed to be a lack of conceptual clarity regarding trauma-related symptoms (e.g., VT, STS, and CF). Some professionals used CF as synonymous to STS, while others use the term to describe a broad range of symptoms that include STS, as well as burnout. In addition, some studies reported on VT-related outcomes, using only general mental health outcomes. As described above, conceptualizing VT-related symptoms can be challenging, considering that the symptoms are multidimensional and may overlap (Newell & MacNeil, 2010; Rauvola et al., 2019; Sprang et al., 2019). However, the use of measurements without consideration for the different underlying definitions VT-related symptoms can lead to unclear outcomes. In this context, we strongly agree with Molnar et al.'s (2017) suggestion that future research should focus on empirically disentangling and operationalizing the concepts of VT, STS, and CF, so that assessment tools can be tailored to be sensitive to these distinctions (Molnar et al., 2017).

Furthermore, no single instrument to assess fully the multidimensional domains of VT-related symptoms is currently available (Molnar et al., 2017; Sprang et al., 2019). Even though existing measurements such as the STSS and ProQOL-5 Scale have shown high validity and reliability in previous studies (Sprang et al., 2019), the scales do not seem to cover VT-related cognitive change, a core concept of VT. Thus, the development of comprehensive validated measures to assess VT-related symptoms is necessary (Branson, 2019). Such measures are essential to identify underlying mechanisms of VT that can serve as targets for VT interventions (Sprang et al., 2019).

Given the limitations of the reviewed research’s study designs described above, we echo and underscore others’ call for rigorous research including randomized design, long-term follow-up to investigate the effects of interventions aimed at preventing and/or ameliorating VT (Bercier & Maynard, 2015; Sprang et al., 2019). Furthermore, our review of qualitative studies showed that many did not specify how they tried to prevent threats to quality and ensure trustworthiness (Lietz & Zayas, 2010). Given that qualitative findings can provide significant contributions in the assessment of participants’ cognitive and emotional changes, future qualitative research should use rigorous approaches and report on efforts to ensure study quality.

Lastly, we found that the existing interventions we reviewed were largely focused on health care settings dealing with disease and grief and loss; only a small number of interventions were developed for health and human service providers serving violence victims. Given that many studies have identified that service providers working with violence and trauma are at higher risk of VT-related symptoms (Baum, 2016; Cohen & Collens, 2013; Raunick et al., 2015), research conducted in settings that offer services to victims of violence should be a priority.

### Limitations

Like all studies, the review has limitations. Given that VT is a multidimensional concept, we included a number of search terms in order to comprehensively search for related articles. However, our search terms might not have covered all of the existing literature on VT-related interventions. In addition, we only focused on VT interventions in health and human service settings, focusing especially on those interventions geared to health and mental health providers. This left out other potentially affected professions, including animal service providers and first responders. We also limited our review to peer-reviewed literature, excluding gray literature and non-peer-reviewed websites. Given the sizable number of peer-reviewed studies concerning the review topic as well as research questions that guided this review, we focused our efforts on the peer-reviewed literature. Nonetheless, the practice and gray literatures may also hold important insights concerning VT that could be explored in future efforts.

## Conclusion

The scoping review shows that VT interventions have been implemented across diverse settings with promising outcomes. However, this review suggests that VT interventions should be tailored to specific service settings and study participants’ diverse backgrounds. Furthermore, there is a need for methodologically robust studies to identify effective VT treatment interventions. Researchers have also suggested that VT offers potential positive aspects by providing an opportunity for increased professional growth and resilience, through—for example—vicarious post-traumatic growth (Arnold et al., 2005), vicarious resilience (Hernández et al., 2007), and shared resilience in a traumatic reality (Nuttman-Shwartz, 2015). We acknowledge that such professional resilience and growth through exposure to trauma can be achieved when the VT is appropriately addressed through effective intervention programs. Thus, developing and implementing VT interventions, based on tailored approaches, should be a primary and essential task, in order to support service providers in pursuing the pathways from VT to professional resilience and growth.

### Summary of Implications for Research, Practice, and Policy

#### Implications for research

- Future studies are needed to clearly define VT-related symptoms and assess the efficacy of interventions, using appropriate measures for specific VT-related symptoms.
- The development of comprehensive validated measures to assess VT-related symptoms is necessary to identify underlying mechanisms of VT that can serve as targets for VT interventions.
- Rigorous research to investigate the effects of VT intervention should be conducted using randomized research designs, long-term follow-up data collection, as well as strategies to increase the robustness and trustworthiness of research, including in the case of qualitative studies.
- Given that studies have identified that service providers working with violence and trauma are at high risk of VT-related symptoms, as a priority for future research, we call on researchers to conduct intervention research in settings that offer services to victims of violence.

#### Implications for practice and policy

- VT interventions need to address specific effects of VT symptoms depending on service setting, type of trauma, and service providers’ individual backgrounds and characteristics.
- VT intervention programs must be designed with clear program targets and goals, including whether they seek to offer preventative interventions or ameliorative treatments.
- In addition to individual-level approaches, primary prevention on an organizational level is necessary to structurally and contextually lower the risk of potential VT among service providers.
- Funders and policymakers should encourage program development and evaluation. In addition, funders need to provide sufficient resources for the rigorous evaluation of VT interventions.
